# Bis(acetato-κ*O*)[1,2-bis­(2-pyridylmeth­oxy)benzene-κ^4^ *N*,*O*,*O*′,*N*′]copper(II) monohydrate

**DOI:** 10.1107/S160053681001874X

**Published:** 2010-05-22

**Authors:** Shuang Zhang, Yu-Jie Wang, Dong-Sheng Ma, Ying Liu, Jin-Sheng Gao

**Affiliations:** a Engineering Research Center of Pesticide of Heilongjiang Province, Heilongjiang University, Harbin 150080, People’s Republic of China; b College of Chemistry and Materials Science, Heilongjiang University, Harbin 150080, People’s Republic of China

## Abstract

In the title compound, [Cu(CH_3_COO)_2_(C_18_H_16_N_2_O_2_)]·H_2_O, the Cu^II^ ion is six-coordinated in a typically Jahn–Teller distorted octa­hedral environment defined by two O and two N atoms from the ligand and two O atoms from acetate anions. A linear chain structure propagating in [010] is built up by inter­molecular O—H⋯O hydrogen bonds involving the uncoordinated water mol­ecules.

## Related literature

For the synthesis and for general backround to flexible pyridyl-based ligands, see: Liu *et al.* (2010*a* ▶,*b* ▶).
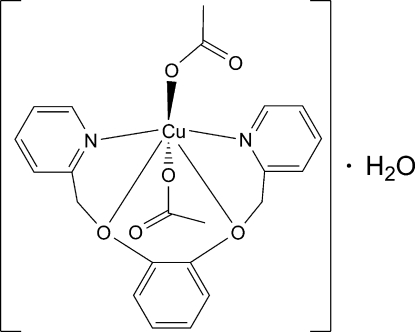


## Experimental

### 

#### Crystal data

[Cu(C_2_H_3_O_2_)_2_(C_18_H_16_N_2_O_2_)]·H_2_O
*M* *_r_* = 491.98
Monoclinic, 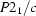

*a* = 11.661 (3) Å
*b* = 14.689 (6) Å
*c* = 15.553 (4) Å
β = 123.540 (11)°
*V* = 2220.5 (12) Å^3^
*Z* = 4
Mo *K*α radiation
μ = 1.03 mm^−1^
*T* = 291 K
0.20 × 0.19 × 0.18 mm

#### Data collection

Rigaku R-AXIS RAPID diffractometer
Absorption correction: multi-scan (*ABSCOR*; Higashi, 1995 ▶) *T*_min_ = 0.818, *T*_max_ = 0.838
21143 measured reflections
4960 independent reflections
3854 reflections with *I* > 2σ(*I*)
*R*_int_ = 0.043

#### Refinement

*R*[*F* ^2^ > 2σ(*F* ^2^)] = 0.034
*wR*(*F* ^2^) = 0.089
*S* = 1.05
4960 reflections
291 parameters
H-atom parameters constrained
Δρ_max_ = 0.28 e Å^−3^
Δρ_min_ = −0.28 e Å^−3^

### 

Data collection: *RAPID-AUTO* (Rigaku, 1998 ▶); cell refinement: *RAPID-AUTO*; data reduction: *CrystalClear* (Rigaku/MSC, 2002 ▶); program(s) used to solve structure: *SHELXS97* (Sheldrick, 2008 ▶); program(s) used to refine structure: *SHELXL97* (Sheldrick, 2008 ▶); molecular graphics: *SHELXTL* (Sheldrick, 2008 ▶); software used to prepare material for publication: *SHELXL97*.

## Supplementary Material

Crystal structure: contains datablocks I, global. DOI: 10.1107/S160053681001874X/ng2778sup1.cif

Structure factors: contains datablocks I. DOI: 10.1107/S160053681001874X/ng2778Isup2.hkl

Additional supplementary materials:  crystallographic information; 3D view; checkCIF report

## Figures and Tables

**Table 1:** Selected bond lengths (Å)

Cu1—O5	1.9529 (16)
Cu1—O3	1.9571 (16)
Cu1—N1	2.0580 (18)
Cu1—N2	2.0823 (18)
Cu1—O2	2.4719 (15)
Cu1—O1	2.5353 (16)

**Table 2:** Hydrogen-bond geometry (Å, °)

*D*—H⋯*A*	*D*—H	H⋯*A*	*D*⋯*A*	*D*—H⋯*A*
O7—H71⋯O4^i^	0.85	1.92	2.772 (3)	174
O7—H72⋯O5	0.85	2.14	2.986 (2)	178

Symmetry code: (i) 
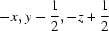
.

**Fig. 1.**
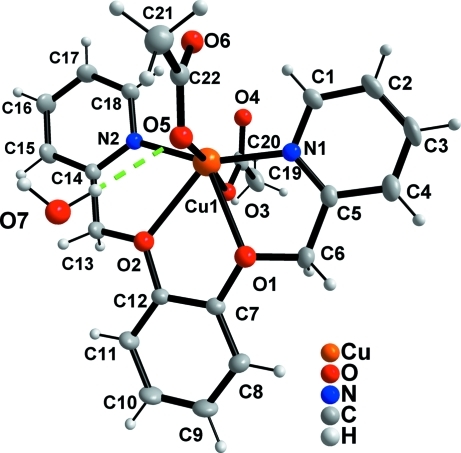
The molecular structure of the title compound, showing displacement ellipsoids at the 30% probability level for non-H atoms.

**Fig. 2.**
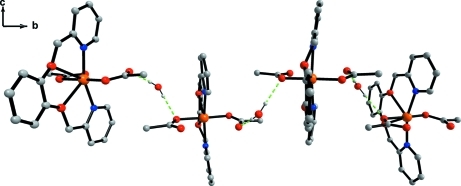
A partial packing view, showing the one-dimensional hydrogen bonding structure. Dashed lines indicate the hydrogen bonds, no involving H atoms have been omitted.

## supplementary crystallographic information

### Comment

N-Heterocyclic ligands coordinated with transition metal ions can form a variety of topology structures, including macrocycles, polyhedra and linear and helical polymers. Our group has report three kinds of flexible pyridyl-based ligands in previous reports (Liu *et al.* 20010a; Liu *et al.* 20010 b). As a part of our continuing work for bipyridyl aromatic ligands, we report the crystal structure of the title compound here.

1,2-Bis(pyridin-2-ylmethoxy)benzene molecule act as a chelating ligand to coordinate with Cu^II^ ion forming a discrete strucutre. Two acetate counter ions also coordinate to the center Cu^II^ ion, resulting the Cu^II^ ion is six-coordinated in quadrangular bipyramid geometry (Figure 1, Table 1).

A one-dimensional chain structure is built up by intermolecular hydrogen bonds involving the uncoordinated water molecules (Figure 2, Table 2).

### Experimental

The 1,2-Bis(pyridin-2-ylmethoxy)benzene was synthesized by the reaction of ο-dihydroxybenzene and 2-chloromethylpyridine hydrochloride under nitrogen atmosphere and alkaline condition (Liu *et al.*, 2010a). Title ligand (0.58 g, 2 mmol) and Cu(CH_3_COO)_2_.H_2_O (0.40 g, 2 mmol) were dissolved in 15 mL e thanol, and then the mixture keep stirring for 30 minute. The resulting solution was filtered, and the filtrate was allowed to stand in a desiccator at room temperature for several days. Bule needle crystals were obtained with yield 34 %.

### Refinement

H atoms bound to C atoms were placed in calculated positions and treated as riding on their parent atoms, with C—H = 0.93 Å (aromatic C), C—H = 0.97 Å (methene C), C—H = 0.98 Å (methyl C), and with *U*_iso_(H) = 1.2*U*_eq_(C). Water H atoms were initially located in a difference Fourier map but they were treated as riding on their parent atoms with O—H = 0.85 Å, and with *U*_iso_(H) = 1.5*U*_eq_(O).

### Crystal data

**Table:**

[Cu(C_2_H_3_O_2_)_2_(C_18_H_16_N_2_O_2_)]·H_2_O	*F*(000) = 1020
*M**_r_* = 491.98	*D*_x_ = 1.472 Mg m^−^^3^
Monoclinic, *P*2_1_/*c*	Mo *K*α radiation, λ = 0.71073 Å
Hall symbol: -P 2ybc	Cell parameters from 15798 reflections
*a* = 11.661 (3) Å	θ = 3.0–27.5°
*b* = 14.689 (6) Å	µ = 1.03 mm^−^^1^
*c* = 15.553 (4) Å	*T* = 291 K
β = 123.540 (11)°	Block, blue
*V* = 2220.5 (12) Å^3^	0.20 × 0.19 × 0.18 mm
*Z* = 4	

### Data collection

**Table:**

Rigaku R-AXIS RAPID diffractometer	4960 independent reflections
Radiation source: fine-focus sealed tube	3854 reflections with *I* > 2σ(*I*)
graphite	*R*_int_ = 0.043
ω scan	θ_max_ = 27.5°, θ_min_ = 3.0°
Absorption correction: multi-scan (*ABSCOR*; Higashi, 1995)	*h* = −14→14
*T*_min_ = 0.818, *T*_max_ = 0.838	*k* = −19→19
21143 measured reflections	*l* = −19→18

### Refinement

**Table:**

Refinement on *F*^2^	Primary atom site location: structure-invariant direct methods
Least-squares matrix: full	Secondary atom site location: difference Fourier map
*R*[*F*^2^ > 2σ(*F*^2^)] = 0.034	Hydrogen site location: inferred from neighbouring sites
*wR*(*F*^2^) = 0.089	H-atom parameters constrained
*S* = 1.05	*w* = 1/[σ^2^(*F*_o_^2^) + (0.0469*P*)^2^ + 0.2754*P*] where *P* = (*F*_o_^2^ + 2*F*_c_^2^)/3
4960 reflections	(Δ/σ)_max_ = 0.001
291 parameters	Δρ_max_ = 0.28 e Å^−^^3^
0 restraints	Δρ_min_ = −0.28 e Å^−^^3^

### Special details

**Table:**

Geometry. All esds (except the esd in the dihedral angle between two l.s. planes) are estimated using the full covariance matrix. The cell esds are taken into account individually in the estimation of esds in distances, angles and torsion angles; correlations between esds in cell parameters are only used when they are defined by crystal symmetry. An approximate (isotropic) treatment of cell esds is used for estimating esds involving l.s. planes.
Refinement. Refinement of *F*^2^ against ALL reflections. The weighted *R*-factor wR and goodness of fit *S* are based on *F*^2^, conventional *R*-factors *R* are based on *F*, with *F* set to zero for negative *F*^2^. The threshold expression of *F*^2^ > σ(*F*^2^) is used only for calculating *R*-factors(gt) etc. and is not relevant to the choice of reflections for refinement. *R*-factors based on *F*^2^ are statistically about twice as large as those based on *F*, and *R*- factors based on ALL data will be even larger.

### Fractional atomic coordinates and isotropic or equivalent isotropic displacement parameters (Å^2^)

**Table:**

	*x*	*y*	*z*	*U*_iso_*/*U*_eq_	
Cu1	0.19750 (2)	0.638427 (17)	0.351821 (17)	0.02824 (9)	
O1	0.43387 (14)	0.58511 (11)	0.40262 (11)	0.0360 (3)	
O2	0.23187 (14)	0.59585 (11)	0.21381 (11)	0.0379 (4)	
O3	0.26556 (16)	0.75769 (10)	0.34343 (12)	0.0394 (4)	
O4	0.13698 (19)	0.81284 (13)	0.39686 (14)	0.0531 (5)	
O5	0.15262 (15)	0.51145 (10)	0.35769 (11)	0.0352 (3)	
O6	0.00215 (19)	0.56093 (13)	0.39381 (14)	0.0533 (5)	
N1	0.33015 (19)	0.64394 (11)	0.50978 (13)	0.0319 (4)	
N2	0.01307 (18)	0.65453 (12)	0.20902 (13)	0.0315 (4)	
C1	0.2732 (3)	0.66269 (16)	0.56418 (17)	0.0400 (5)	
H1	0.1779	0.6651	0.5294	0.048*	
C2	0.3524 (3)	0.67823 (18)	0.66939 (19)	0.0513 (6)	
H2	0.3115	0.6921	0.7049	0.062*	
C3	0.4931 (3)	0.6726 (2)	0.72009 (18)	0.0585 (7)	
H3	0.5486	0.6836	0.7907	0.070*	
C4	0.5530 (3)	0.65064 (18)	0.66613 (18)	0.0503 (6)	
H4	0.6479	0.6443	0.7003	0.060*	
C5	0.4679 (2)	0.63844 (15)	0.56047 (16)	0.0347 (5)	
C6	0.5334 (2)	0.62142 (17)	0.50060 (17)	0.0400 (5)	
H6A	0.5692	0.6780	0.4924	0.048*	
H6B	0.6093	0.5790	0.5381	0.048*	
C7	0.4697 (2)	0.58313 (15)	0.33102 (16)	0.0336 (5)	
C8	0.6037 (2)	0.57611 (17)	0.35554 (19)	0.0432 (6)	
H8	0.6772	0.5743	0.4242	0.052*	
C9	0.6276 (3)	0.57171 (19)	0.2769 (2)	0.0532 (7)	
H9	0.7170	0.5673	0.2929	0.064*	
C10	0.5183 (3)	0.57400 (19)	0.1761 (2)	0.0525 (7)	
H10	0.5343	0.5704	0.1238	0.063*	
C11	0.3831 (3)	0.58167 (17)	0.15022 (19)	0.0440 (6)	
H11	0.3098	0.5833	0.0814	0.053*	
C12	0.3594 (2)	0.58687 (14)	0.22831 (16)	0.0325 (5)	
C13	0.1171 (2)	0.61228 (17)	0.11292 (16)	0.0387 (5)	
H13A	0.0978	0.5588	0.0704	0.046*	
H13B	0.1364	0.6627	0.0825	0.046*	
C14	−0.0064 (2)	0.63471 (15)	0.11764 (16)	0.0328 (4)	
C15	−0.1366 (2)	0.63615 (18)	0.02673 (17)	0.0467 (6)	
H15	−0.1481	0.6222	−0.0359	0.056*	
C16	−0.2490 (3)	0.65856 (19)	0.0306 (2)	0.0526 (7)	
H16	−0.3370	0.6584	−0.0293	0.063*	
C17	−0.2301 (2)	0.68105 (18)	0.12371 (19)	0.0454 (6)	
H17	−0.3041	0.6975	0.1275	0.054*	
C18	−0.0983 (2)	0.67845 (16)	0.21095 (17)	0.0369 (5)	
H18	−0.0849	0.6937	0.2739	0.044*	
C19	0.2749 (4)	0.91611 (19)	0.3717 (2)	0.0691 (9)	
H19A	0.3666	0.9223	0.4319	0.104*	
H19B	0.2168	0.9618	0.3728	0.104*	
H19C	0.2763	0.9235	0.3110	0.104*	
C20	0.2198 (3)	0.82288 (17)	0.37095 (16)	0.0406 (5)	
C21	0.0351 (3)	0.40102 (19)	0.3948 (2)	0.0531 (6)	
H21A	−0.0609	0.3877	0.3477	0.080*	
H21B	0.0603	0.3919	0.4643	0.080*	
H21C	0.0887	0.3614	0.3811	0.080*	
C22	0.0622 (2)	0.49872 (16)	0.38126 (16)	0.0368 (5)	
O7	0.1077 (2)	0.40557 (15)	0.17760 (17)	0.0729 (6)	
H71	0.0314	0.3772	0.1505	0.109*	
H72	0.1223	0.4355	0.2295	0.109*	

### Atomic displacement parameters (Å^2^)

**Table:**

	*U*^11^	*U*^22^	*U*^33^	*U*^12^	*U*^13^	*U*^23^
Cu1	0.02986 (14)	0.03193 (14)	0.02291 (13)	−0.00047 (11)	0.01457 (11)	0.00016 (10)
O1	0.0302 (7)	0.0476 (10)	0.0287 (8)	−0.0046 (7)	0.0153 (7)	−0.0046 (7)
O2	0.0285 (7)	0.0602 (11)	0.0254 (7)	0.0034 (7)	0.0152 (7)	−0.0004 (7)
O3	0.0487 (9)	0.0370 (9)	0.0358 (8)	−0.0042 (7)	0.0254 (8)	−0.0001 (7)
O4	0.0606 (11)	0.0524 (11)	0.0561 (11)	0.0162 (9)	0.0384 (10)	0.0136 (9)
O5	0.0353 (8)	0.0358 (8)	0.0345 (8)	−0.0041 (6)	0.0193 (7)	−0.0006 (6)
O6	0.0604 (11)	0.0581 (12)	0.0542 (11)	0.0101 (9)	0.0397 (10)	0.0089 (9)
N1	0.0400 (10)	0.0301 (9)	0.0247 (8)	−0.0018 (8)	0.0173 (8)	0.0000 (7)
N2	0.0314 (9)	0.0360 (10)	0.0272 (9)	0.0014 (7)	0.0163 (8)	0.0017 (7)
C1	0.0528 (14)	0.0394 (13)	0.0335 (12)	−0.0003 (10)	0.0274 (12)	0.0016 (9)
C2	0.0784 (19)	0.0500 (15)	0.0348 (13)	−0.0035 (14)	0.0372 (14)	−0.0017 (11)
C3	0.077 (2)	0.0650 (18)	0.0212 (11)	−0.0089 (15)	0.0193 (13)	−0.0039 (11)
C4	0.0500 (15)	0.0558 (17)	0.0300 (12)	−0.0082 (12)	0.0125 (12)	0.0019 (11)
C5	0.0396 (12)	0.0301 (11)	0.0275 (10)	−0.0034 (9)	0.0141 (10)	0.0020 (9)
C6	0.0328 (11)	0.0457 (14)	0.0327 (11)	−0.0070 (10)	0.0126 (10)	−0.0034 (10)
C7	0.0347 (11)	0.0347 (12)	0.0363 (12)	0.0012 (9)	0.0226 (10)	0.0000 (9)
C8	0.0326 (12)	0.0491 (15)	0.0467 (14)	0.0044 (10)	0.0213 (12)	0.0004 (11)
C9	0.0452 (14)	0.0641 (18)	0.0663 (18)	0.0055 (12)	0.0408 (15)	−0.0027 (14)
C10	0.0540 (15)	0.0651 (18)	0.0588 (17)	0.0042 (13)	0.0439 (15)	−0.0010 (13)
C11	0.0454 (13)	0.0550 (16)	0.0400 (13)	0.0024 (11)	0.0288 (12)	−0.0016 (11)
C12	0.0327 (11)	0.0340 (12)	0.0354 (11)	0.0003 (9)	0.0216 (10)	−0.0022 (9)
C13	0.0365 (12)	0.0523 (14)	0.0260 (11)	0.0034 (10)	0.0164 (10)	0.0011 (9)
C14	0.0341 (11)	0.0356 (12)	0.0274 (10)	0.0001 (9)	0.0163 (10)	0.0012 (9)
C15	0.0424 (13)	0.0627 (17)	0.0261 (11)	0.0087 (12)	0.0133 (11)	0.0013 (11)
C16	0.0332 (12)	0.074 (2)	0.0350 (13)	0.0103 (12)	0.0089 (11)	0.0045 (12)
C17	0.0366 (12)	0.0519 (15)	0.0456 (14)	0.0120 (11)	0.0215 (12)	0.0096 (11)
C18	0.0376 (12)	0.0398 (13)	0.0344 (11)	0.0070 (10)	0.0206 (11)	0.0040 (10)
C19	0.116 (3)	0.0406 (16)	0.0568 (17)	−0.0208 (16)	0.0513 (19)	−0.0090 (13)
C20	0.0542 (15)	0.0365 (13)	0.0269 (11)	0.0013 (11)	0.0199 (11)	0.0057 (9)
C21	0.0548 (15)	0.0511 (16)	0.0530 (16)	−0.0142 (13)	0.0295 (14)	0.0008 (12)
C22	0.0347 (11)	0.0441 (13)	0.0277 (11)	−0.0024 (10)	0.0146 (10)	0.0012 (9)
O7	0.0755 (14)	0.0811 (16)	0.0852 (15)	−0.0315 (12)	0.0590 (14)	−0.0353 (12)

### Geometric parameters (Å, °)

**Table:**

Cu1—O5	1.9529 (16)	C7—C8	1.393 (3)
Cu1—O3	1.9571 (16)	C8—C9	1.398 (3)
Cu1—N1	2.0580 (18)	C8—H8	0.9300
Cu1—N2	2.0823 (18)	C9—C10	1.370 (4)
Cu1—O2	2.4719 (15)	C9—H9	0.9300
Cu1—O1	2.5353 (16)	C10—C11	1.401 (3)
O1—C7	1.389 (2)	C10—H10	0.9300
O1—C6	1.414 (3)	C11—C12	1.389 (3)
O2—C12	1.381 (2)	C11—H11	0.9300
O2—C13	1.411 (3)	C13—C14	1.519 (3)
O3—C20	1.280 (3)	C13—H13A	0.9700
O4—C20	1.244 (3)	C13—H13B	0.9700
O5—C22	1.307 (3)	C14—C15	1.390 (3)
O6—C22	1.231 (3)	C15—C16	1.385 (3)
N1—C5	1.345 (3)	C15—H15	0.9300
N1—C1	1.362 (3)	C16—C17	1.378 (3)
N2—C14	1.341 (3)	C16—H16	0.9300
N2—C18	1.362 (3)	C17—C18	1.378 (3)
C1—C2	1.383 (3)	C17—H17	0.9300
C1—H1	0.9300	C18—H18	0.9300
C2—C3	1.376 (4)	C19—C20	1.510 (3)
C2—H2	0.9300	C19—H19A	0.9600
C3—C4	1.394 (4)	C19—H19B	0.9600
C3—H3	0.9300	C19—H19C	0.9600
C4—C5	1.384 (3)	C21—C22	1.509 (3)
C4—H4	0.9300	C21—H21A	0.9600
C5—C6	1.516 (3)	C21—H21B	0.9600
C6—H6A	0.9700	C21—H21C	0.9600
C6—H6B	0.9700	O7—H71	0.8527
C7—C12	1.392 (3)	O7—H72	0.8496
			
O5—Cu1—O3	170.76 (6)	C7—C8—H8	120.0
O5—Cu1—N1	91.48 (6)	C9—C8—H8	120.0
O3—Cu1—N1	88.82 (7)	C10—C9—C8	119.4 (2)
O5—Cu1—N2	90.74 (7)	C10—C9—H9	120.3
O3—Cu1—N2	92.58 (7)	C8—C9—H9	120.3
N1—Cu1—N2	157.06 (7)	C9—C10—C11	121.2 (2)
O5—Cu1—O2	88.58 (6)	C9—C10—H10	119.4
O3—Cu1—O2	84.40 (6)	C11—C10—H10	119.4
N1—Cu1—O2	132.15 (6)	C12—C11—C10	119.3 (2)
N2—Cu1—O2	70.73 (6)	C12—C11—H11	120.3
O5—Cu1—O1	87.92 (6)	C10—C11—H11	120.3
O3—Cu1—O1	83.47 (6)	O2—C12—C11	125.4 (2)
N1—Cu1—O1	70.70 (6)	O2—C12—C7	114.82 (17)
N2—Cu1—O1	132.21 (6)	C11—C12—C7	119.81 (19)
O2—Cu1—O1	61.48 (5)	O2—C13—C14	109.00 (16)
C7—O1—C6	116.14 (16)	O2—C13—H13A	109.9
C7—O1—Cu1	120.87 (12)	C14—C13—H13A	109.9
C6—O1—Cu1	108.85 (12)	O2—C13—H13B	109.9
C12—O2—C13	118.16 (16)	C14—C13—H13B	109.9
C12—O2—Cu1	123.96 (12)	H13A—C13—H13B	108.3
C13—O2—Cu1	114.41 (12)	N2—C14—C15	121.7 (2)
C20—O3—Cu1	112.84 (14)	N2—C14—C13	119.23 (18)
C22—O5—Cu1	115.36 (14)	C15—C14—C13	119.09 (19)
C5—N1—C1	118.86 (19)	C16—C15—C14	119.2 (2)
C5—N1—Cu1	124.32 (14)	C16—C15—H15	120.4
C1—N1—Cu1	116.53 (15)	C14—C15—H15	120.4
C14—N2—C18	118.30 (18)	C17—C16—C15	119.8 (2)
C14—N2—Cu1	125.17 (14)	C17—C16—H16	120.1
C18—N2—Cu1	116.19 (14)	C15—C16—H16	120.1
N1—C1—C2	122.2 (2)	C18—C17—C16	118.2 (2)
N1—C1—H1	118.9	C18—C17—H17	120.9
C2—C1—H1	118.9	C16—C17—H17	120.9
C3—C2—C1	118.3 (2)	N2—C18—C17	122.9 (2)
C3—C2—H2	120.9	N2—C18—H18	118.5
C1—C2—H2	120.9	C17—C18—H18	118.5
C2—C3—C4	120.3 (2)	C20—C19—H19A	109.5
C2—C3—H3	119.9	C20—C19—H19B	109.5
C4—C3—H3	119.9	H19A—C19—H19B	109.5
C5—C4—C3	118.4 (2)	C20—C19—H19C	109.5
C5—C4—H4	120.8	H19A—C19—H19C	109.5
C3—C4—H4	120.8	H19B—C19—H19C	109.5
N1—C5—C4	121.9 (2)	O4—C20—O3	124.2 (2)
N1—C5—C6	119.58 (18)	O4—C20—C19	120.5 (2)
C4—C5—C6	118.5 (2)	O3—C20—C19	115.3 (2)
O1—C6—C5	109.39 (18)	C22—C21—H21A	109.5
O1—C6—H6A	109.8	C22—C21—H21B	109.5
C5—C6—H6A	109.8	H21A—C21—H21B	109.5
O1—C6—H6B	109.8	C22—C21—H21C	109.5
C5—C6—H6B	109.8	H21A—C21—H21C	109.5
H6A—C6—H6B	108.2	H21B—C21—H21C	109.5
O1—C7—C12	115.01 (17)	O6—C22—O5	123.8 (2)
O1—C7—C8	124.8 (2)	O6—C22—C21	120.1 (2)
C12—C7—C8	120.17 (19)	O5—C22—C21	116.0 (2)
C7—C8—C9	120.0 (2)	H71—O7—H72	109.2

### Hydrogen-bond geometry (Å, °)

**Table:**

*D*—H···*A*	*D*—H	H···*A*	*D*···*A*	*D*—H···*A*
O7—H71···O4^i^	0.85	1.92	2.772 (3)	174
O7—H72···O5	0.85	2.14	2.986 (2)	178

Symmetry codes: (i) −*x*, *y*−1/2, −*z*+1/2.
